# Update on the first-line treatment for *Helicobacter pylori* infection - a continuing challenge from an old enemy

**DOI:** 10.1186/s40364-017-0103-x

**Published:** 2017-07-11

**Authors:** Chih-Chieh Huang, Kuo-Wang Tsai, Tzung-Jiun Tsai, Ping-I Hsu

**Affiliations:** 1 Division of Gastroenterology, Department of Internal Medicine, Kaohsiung Veterans General Hospital and National Yang-Ming University, Kaohsiung, Taiwan; 2 Department of Medical Education and Research, Kaohsiung Veterans General Hospital, Kaohsiung, Taiwan; 3 Taiwan Acid-related Disease (TARD) Study Group, Kaohsiung, Taiwan; 4 Division of Gastroenterology, Department of Internal Medicine, Kaoshiung Veterans General Hospital, 386 Ta Chung 1st Road, Kaohsiung, 813 Taiwan, ROC

**Keywords:** *Helicobacter pylori*, First-line, Therapy, Hybrid therapy, Sequential therapy, Concomitant therapy, Triple therapy

## Abstract

Because the prevalence of antibiotic resistance markedly increases with time worldwide, anti-*H. pylori* treatment is continuing to be a great challenge forsphysicians in clinical practice. The Real-world Practice & Expectation of Asia-Pacific Physicians and Patients in *Helicobacter Pylori* Eradication (REAP-HP) Survey demonstrated that the accepted minimal eradication rate of anti-*H. pylori* regimen in *H. pylori*-infected patients was 91%. The Kyoto Consensus Report on *Helicobacter Pylori* Gastritis also recommended that, within any region, only regimens which reliably produce eradication rates of ≥90% in that population should be used for empirical treatment. This article is aimed to review current first-line eradication regimens with a per-protocol eradication rate exceeding 90% in most geographic areas. In regions with low (≦15%) clarithromycin resistance, 14-day hybrid (or reverse hybrid), 10 ~ 14-day sequential, 7 ~ 14-day concomitant, 10 ~ 14-day bismuth quadruple or 14-day triple therapy can achieve a high eradication rate in the first-line treatment of *H. pylori* infection. However, in areas with high (>15%) clarithromycin resistance, standard triple therapy should be abandoned because of low eradication efficacy, and 14-day hybrid (or reverse hybrid), 10 ~ 14-day concomitant or 10 ~ 14-day bismuth quadruple therapy are the recommended regimens. If no recent data of local antibiotic resistances of *H. pylori* strains are available, universal high efficacy regimens such as 14-day hybrid (or reverse hybrid), concomitant or bismuth quadruple therapy can be adopted to meet the recommendation of consensus report and patients’ expectation.

## Background

*Helicobacter pylori* (*H. pylori*) infect more than 50% of humans globally. It is the major cause of chronic gastritis, peptic ulcer, gastric mucosa-associated lymphoid tissue lymphoma (MALToma), and gastric adenocarcinoma [1, 2]. Eradication of *H. pylori* can effectively prevent the recurrence of peptic ulcer disease [3, 4]. Anti- *H. pylori* therapy is currently recommended in the treatment of *H pylori*-related gastric MALToma [5]. Additionally, eradication of *H pylori* is advocated as a preventative method in regions with high incidence of gastric adenocarcinoma [6, 7].

A recent survey for *H. pylori* eradication therapy revealed that 7-day standard triple therapy remained the most popular regimen in the Asia-Pacific region [8]. However, the eradication rate of 7-day standard triple therapy has declined to less than 80% in most countries worldwide owing to increasing resistance rate to antibiotics [9–12]. The Kyoto Consensus Report on *Helicobacter Pylori* Gastritis recommends that, within any region, only regimens which reliably produce eradication rates of ≥90% in that population should be used for empirical treatment [13]. Current medicine practice emphasizes shared decision making with patients. It is therefore important for physicians to know the expectations of patients and try to meet patients’ expectations on eradication therapy when they prescribe anti-*H. pylori* regimen. The survey for the Real-world Practice & Expectation of Asia-Pacific Physicians and Patients in *Helicobacter Pylori* Eradication (REAP-HP) showed that the expected minimal eradication rate in patients was 91.4% [8]. Hence, physicians should prescribe an eradication regimen with cure rate exceeding 90% to treat their *H. pylori*-infected patients. This article is aimed to review current novel first-line eradication regimens with a per-protocol (PP) eradication rate exceeding 90% in most geographic areas.

## Current antibiotic resistance

The main reasons for eradication failure of standard triple therapy include antibiotic resistance, poor compliance and rapid metabolism of proton pump inhibitor (PPI) [10, 14, 15]. Clarithromycin resistance has been identified as the main reason for the failure of standard triple therapy [16, 17]. Pooled data from 20 studies involving 1975 patients treated with standard triple therapy showed an eradication rate of 88% in clarithromycin-sensitive strains versus 18% in clarithromycin-resistant strains [10]. Therefore, the background rate of clarithromycin resistance is critically important for the efficacy of standard triple therapy.

The prevalence of antibiotic resistance varies in different geographic regions and appears to be increasing with time in most countries worldwide [18–24]. For example, an increase in clarithromycin resistance was observed in Korea from 7.0% in 2009 to 16.0% in 2011 [23]. The resistance rate of *H. pylori* to clarithromycin in Japan had increased gradually from 2% in 1996 to approximately 30% in 2004 [22]. In China, a marked increase of clarithromycin resistance was seen from 8.6% in 2000 to 20.7% in 2009 [21]. The recent clarithromycin resistance rates of *H. pylori* in China, Taiwan, Japan, Turkey, Italy and the United States are approximately 50, 15, 30, 40, 30 and 13%, respectively [20]. The prevalence of metronidazole resistance quite varies in different countries. The resistance rate of *H. pylori* to metronidazole remains high in China (41.6 - 67.4% between 2008 and 2015). In contrast, the prevalence of metronidazole resistance is extremely low in Japan [22]. Nishizawa et al. described an overall resistance rate of 2.1% in patients studied from 2012 to 2104 [24]. In Korea, the prevalence of metronidazole resistance had increased from 45.1% in 2009 to 56.3% in 2011 [23]. Besides clarithromycin and metronidazole, amoxicillin is also one of the most commonly used antibiotics in first-line anti-*H. pylori* therapy. However, resistance to amoxicillin remains extremely low (less than 5%) in most countries [16, 20, 23, 24].

## Updated *H. pylori* therapy

**Fig. 1:**
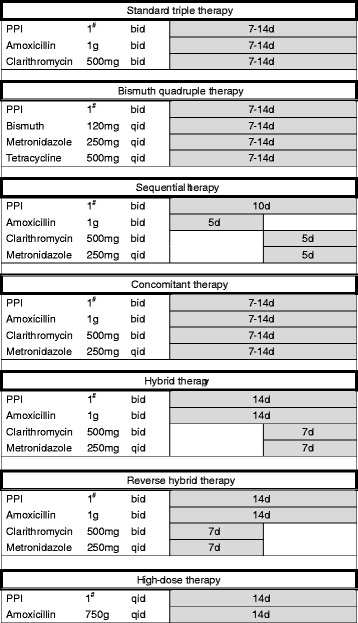
Current anti-*H pylori* regimens

With the rising prevalence of antimicrobial resistance, standard triple therapy is no longer effective in most countries [15–17]. The eradication rate of the 7-day standard triple therapy in 14 hospitals over the Tokyo metropolitan area in 2010 was only 66.5 and 378.7% by intention-to-treat (ITT) and per-protocol (PP) analyses, respectively [25]. The regimen achieved a success rate of 68.5% by PP analysis in Korea [26]. Clinicians should therefore avoid triple therapies unless it has been proven to achieve an adequate success rate locally. Currently, several strategies including bismuth quadruple, non-bismuth quadruple (i.e., sequential, concomitant and hybrid) and high-dose dual therapies have proposed to increase the eradication rate [27–32]. Figure 1 summaries the regimens of these new therapies.

### Sequential therapy

Sequential therapies developed by Zullo et al. consists of a 5-day dual therapy with a PPI (standard dose, b.i.d.) and amoxicillin (1 g, b.i.d.) followed by a 5-day triple therapy with a PPI (standard dose, b.i.d.), clarithromycin (500 mg, b.i.d.) and metronidazole (500 mg, b.i.d.) [27]. Clarithromycin resistance also reduces the efficacy of sequential therapy. However, the impact of clarithromycin resistance on sequential therapy is markedly less than that on standard triple therapy because sequential therapy contains metronidazole as an additional antibiotic [16]. A randomized, double-blind, placebo-controlled trial demonstrated that the PP eradication rates of sequential therapy and standard triple therapy for clarithromycin-resistant strains were 89 and 29%, respectively [33]. The eradication rate of 10-day sequential therapy was superior to that of 7-day standard triple therapy in several randomized controlled trials [16, 33]. However, meta-analysis revealed that 10-day sequential therapy was not superior to 14-day standard triple therapy [34]. Either clarithromycin or metronidazole resistance can undermine the efficacy of sequential therapy [16, 35]. The eradication rates of 10-day sequential therapy for the strains with non-resistance, single clarithromycin resistance, single metronidazole resistance and dual resistances were 95, 70, 78, and 43%, respectively, in a recent prospective randomized controlled trial [35]. In addition, this therapy is more complex and requires changing antibiotics during the treatment course which may reduce the treatment compliance of patients.

### Concomitant therapy

Concomitant therapy is another novel regimen proven successful in the presence of clarithromycin resistance [16, 30]. It is a 4-drug regimen containing a PPI (standard dose, b.i.d.), clarithromycin (500 mg, b.i.d.), amoxicillin (1 g, b.i.d.) and metronidazole (500 mg, b.i.d.) which are all given for the entire duration of therapy (Fig. 1). Meta-analysis demonstrated that concomitant therapy is more effective than standard triple therapy (90% versus 78% by ITT analysis) [36]. Dual clarithromycin and metronidazole resistance undermines the efficacy of concomitant therapy. A recent randomized controlled trial revealed that the eradication rates of 7-day concomitant therapy for the *H pylori* strains with nonresistance, single clarithromycin resistance, single metronidazole resistance, and dual clarithromycin and metronidazole resistance were 100.0, 100.0, 100.0, and 66.7%, respectively [16].

The efficacy of concomitant therapy was also related to the duration of treatment [37]. A tendency toward better results with longer treatments (7–10 days versus 3*–*5 days) has been observed [37]. Concomitant therapy is less complex than sequential therapy as this regimen does not involve changing drugs halfway through.

### Hybrid and reverse hybrid therapies

Hybrid therapy was introduced by Hsu et al. in Taiwan in 2011 [29]. This regimen consists of a dual therapy with a proton pump inhibitor (PPI) and amoxicillin for 7 days followed by a quadruple regimen with a PPI, amoxicillin, clarithromcyin and metronidazole for 7 days. It produced an eradication rate of 99.1% by PP analysis and 97.4% by ITT analysis in Taiwan [16]. A systemic review showed that the eradication rates of hybrid therapy for clarithromycin-sensitive and resistant strains were 99.1 and 85.7%, respectively [38]. The impact of metronidazole resistance on the efficacy of the new therapy also appeared minor (susceptible strains: 100% [68/68]; resistant strains: 94.2% [49/52]) [38]. Several randomized controlled trials demonstrated that hybrid regimens were comparable with or more effective than sequential regimens [38–42]. A recent large multicentre randomized controlled trial demonstrated that both 14-day hybrid and 14-day concomitant therapies cured more than 90% of patients with *H pylori* infections in areas of high clarithromycin and metronidazole resistance [43]. However, significantly more patients were compliant with hybrid therapy (98.8%) than concomitant therapy (95.2%) [43]. Meta-analysis revealed that hybrid therapy and concomitant therapy achieved comparable eradication rate [38]. Recently, the Taiwan *H pylori* Consensus Report recommends 14-day hybrid, concomitant and bismuth quadruple therapies as choices of anti-*H pylori* treatment in areas with either high or low clarithromycin resistance [44].

Switching drugs halfway through the course increases the complexity of an anti-*H pylori* regimen. Reversing the sequence of drug administration (a quadruple regimen followed by a dual regimen) may simplify hybrid therapy (a one-step two-phase therapy; Fig. 1). A pilot multicenter, randomized trial demonstrated that reverse hybrid therapy was highly effective in Taiwan (eradication rate by PP analysis: 96%) and superior to standard triple therapy [43]. A retrospective cohort study demonstrated that reverse hybrid therapy yielded a similar eradication rate as standard hybrid therapy [45]. The new therapy appears to be a simple, highly effective, and well-tolerated treatment for *H pylori* infection in the era of increasing antibiotic resistance.

### High-dose dual therapy

High-dose dual therapy developed by Yang et al. is another emerging treatment for *H pylori* infection [32]. The new therapy consists of high-dose PPI (one tablet q.i.d) and amoxicillin (750 mg q.i.d), which may keep the intragastric pH at a value higher than 6.5 regardless of *CYP2C19* genotype [46] and maintain steady plasma concentration of amoxicillin above the minimal inhibitory concentration for *H pylori* [47]. The efficacy of the new therapy was significantly higher than that of standard triple therapy in Taiwan [42]. However, it was less effective as the first-line therapy for eradicating *H pylori* in Korea [48]. Currently, data on high-dose dual therapy are scarce, and further studies to investigate the simple regimens are needed.

### Bismuth quadruple therapy

Bismuth-containing quadruple therapy containing a PPI, bismuth, metronidazole and tetracycline is recommended as the choice treatment for *H pylori* infection in areas of either low or high clarithromycin resistance in the Maastricht V/Florence Consensus Report [49]. The optimal treatment duration of bismuth-containing quadruple therapy remains unclear. However, the efficacy of bismuth quadruple therapy for 1–3 days, 4 days or 7 days was less effective than when given for 10–14 days [50]. Bismuth quadruple therapy for 10–14 days achieved ≧ 85% eradication rate, even in areas with a high prevalence of metronidazole resistance [50]. However, 14-day bismuth quadruple therapy had a much higher frequency of adverse events than 14-day hybrid therapy (55.5% vs 15.7%, *P* < 0.001) [51].

### Quinolone-containing triple therapy

A quinolone-containing triple therapy is effective as the first-line therapy for *H pylori* infection. Its cure rates range from 72 to 96% [52]. The regimen might be considered in populations with clarithromycin resistance greater than 15–20% and quinolone resistance less than 10% [53]. Nonetheless, a quinolone-based triple therapy is not generally recommended as a first-line therapy due to concerns of the rising prevalence of quinolone-resistant strains. Additionally, the increased use of quinolone would likely lead to the development of more quinolone-resistant pathogens for respiratory and urogenital tract infection.

## Conclusion

**Fig. 2:**
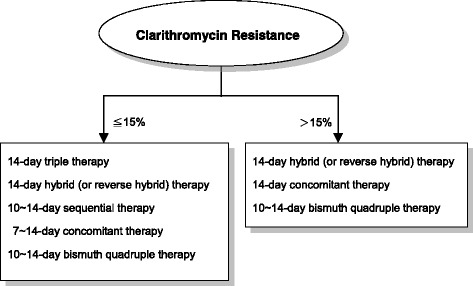
Recommended anti-*H pylori* regimens in regions with different clarithromycin resistant rates

Current consensus report on *H. pylori* eradication therapy recommends that only regimens that reliably produce eradication rates of ≥90% should be used for empirical treatment. The REAP-HP Survey also revealed that the accepted minimal eradication rate of anti-*H. pylori* therapy in infected patients was 91%. To meet the recommendation of the consensus report and patients’ expectation, 14-day triple, 14-day hybrid (or reverse hybrid), 10 ~ 14-day sequential, 7 ~ 14-day concomitant or 10 ~ 14-day bismuth quadruple therapy can be adopted as the first-line treatment for *H. pylori* infection in areas with low (≦15%) clarithromycin resistance (Fig. 2). In areas with high (>15%) clarithromycin resistance, standard triple therapy should be abandoned, and 14-day hybrid (or reverse hybrid), 10 ~ 14-day concomitant or 10 ~ 14-day bismuth quadruple therapy can be used to treat *H. pylori* infection. If no recent data of local antibiotic resistances of *H. pylori* strains are available, universal high efficacy regimens such as 14-day hybrid (or reverse hybrid), concomitant or bismuth quadruple therapy can be adopted to meet the recommendation of consensus report and patients’ expectation.

## Abbreviations

*H. pylori*: *Helicobacter pylori*
ITT: Intention to treat
MALToma: Mucosa-associated lymphoid tissue lymphoma
PP: Per protocol
PPI: Proton pump inhibitor
REAP-HP: Real-world practice & expectation of Asia-Pacific physicians and patients in *Helicobacter pylori* eradication
